# Factors influencing long‐term survival after cytoreductive surgery and hyperthermic intraperitoneal chemotherapy for pseudomyxoma peritonei originating from appendiceal neoplasms

**DOI:** 10.1002/bjs5.50134

**Published:** 2019-02-19

**Authors:** W. J. van Eden, N. F. M. Kok, P. Snaebjornsson, K. Jóźwiak, K. Woensdregt, P. D. Bottenberg, H. Boot, A. G. J. Aalbers

**Affiliations:** ^1^ Department of Surgical Oncology the Netherlands Cancer Institute Amsterdam the Netherlands; ^2^ Department of Pathology the Netherlands Cancer Institute Amsterdam the Netherlands; ^3^ Department of Epidemiology and Biostatistics the Netherlands Cancer Institute Amsterdam the Netherlands; ^4^ Medical Oncology and Gastroenterology the Netherlands Cancer Institute Amsterdam the Netherlands

## Abstract

**Background:**

Pseudomyxoma peritonei (PMP) is a rare disease, most commonly of appendiceal origin. Treatment consists of cytoreductive surgery and hyperthermic intraperitoneal chemotherapy (CRS–HIPEC). The aim of this study was to identify prognostic factors for recurrence and survival.

**Methods:**

This was an observational study using a prospectively designed database containing consecutive patients with PMP originating from the appendix, undergoing CRS–HIPEC at a tertiary referral centre between 1996 and 2015. Histopathological slides were reassessed. Cox regression was used for multivariable analyses.

**Results:**

Of 225 patients identified, 36 (16·0 per cent) were diagnosed with acellular mucin, 149 (66·2 per cent) had disseminated peritoneal adenomucinosis (DPAM) and 40 (17·8 per cent) had peritoneal mucinous carcinomatosis (PMCA). The 5‐year overall survival (OS) rates were 93, 69·8 and 55 per cent respectively. Recurrence was observed in 120 patients (53·3 per cent), 39 of whom (17·3 per cent) were treated with a second CRS–HIPEC procedure. Factors independently associated with poor disease‐free survival were six or seven affected regions (hazard ratio (HR) 6·01, 95 per cent c.i. 2·04 to 17·73), incomplete cytoreduction (R2a resection: HR 1·67, 1·05 to 2·65; R2b resection: HR 2·00, 1·07 to 3·73), and more than threefold raised carcinoembryonic antigen (CEA) and/or carbohydrate antigen (CA) 19‐9 level (HR 2·31, 1·30 to 4·11). Factors independently associated with poorer OS were male sex (HR 1·74, 1·09 to 2·77), incomplete cytoreduction (R2a resection: HR 1·87, 1·14 to 3·08; R2b resection: HR 2·28, 1·19 to 4·34), and more than threefold raised CEA and/or CA19‐9 level (HR 2·89, 1·36 to 6·16).

**Conclusion:**

CEA and CA19‐9 levels raised more than threefold above the upper limit identify patients with PMP of appendiceal origin and poorer survival.

## Introduction

Pseudomyxoma peritonei (PMP) is a rare disease with an incidence of 1–2 per million per year^1^, ^2^. PMP frequently originates from appendiceal lesions. Rarer origins include mucinous colorectal adenocarcinomas or mucinous ovarian tumours^3^, ^4^. Traditionally PMP was treated with repeated cytoreductive surgery (CRS) only, but this has been gradually replaced by CRS combined with hyperthermic intraperitoneal chemotherapy (HIPEC)^4^, ^5^, ^6^. After treatment with CRS–HIPEC, the 10‐year overall survival (OS) rate is 50–60 per cent^4^, ^5^, ^7^. Although CRS–HIPEC is a major surgical procedure that significantly impacts quality of life, many patients seem willing to undergo further CRS–HIPEC if required^8^, ^9^.

PMP is a clinicopathological syndrome with mucinous ascites, including neoplastic mucinous epithelium within the peritoneal cavity^10^. Most patients have symptoms at the time of initial diagnosis, with increased abdominal girth, abdominal discomfort, nausea, vomiting or weight loss^11^. Clinical symptoms are associated with poorer survival^11^. Other prognostic factors include serum tumour markers and histological findings. Serum tumour marker levels appeared to have a diagnostic and prognostic value in patients with PMP^1^, ^12^. Levels of carcinoembryonic antigen (CEA), carbohydrate antigen (CA) 19‐9 and cancer antigen 125 (CA125) may be raised in these patients. Some studies^13^, ^14^, ^15^ have shown that increase in one or more of these tumour markers may be associated with worse prognosis.

PMP is classified histopathologically as acellular mucin, disseminated peritoneal adenomucinosis (DPAM) or peritoneal mucinous carcinomatosis (PMCA)^16^. Acellular mucin has been associated with the best prognosis^17^. The aim of this study was to obtain more insight into the clinical course and management of PMP originating from appendiceal neoplasms in patients treated with CRS–HIPEC. Histopathology was reassessed to better determine independent prognostic factors, based on the 20‐year experience in the treatment of PMP at a single institution.

## Methods

### Patients and setting

This was an observational study performed in the Netherlands Cancer Institute, a tertiary oncology referral centre in Amsterdam. Data from consecutive patients who underwent CRS–HIPEC were entered in a prospectively developed database. Patients diagnosed with PMP originating from a mucinous appendiceal neoplasm who were treated with CRS–HIPEC between January 1996 and December 2015 were included in the study. PMP was defined as a clinical entity consisting of mucinous ascites. Patients were excluded if they had received extra‐abdominal treatment for PMP or had PMP with a primary origin in ovarian or colorectal carcinoma, or an unknown primary location. PMP was diagnosed either clinically or radiologically, and confirmed histologically.

This study received approval of the Translational Research Board of the Netherlands Cancer Institute (study number CFMPB515). All data were processed anonymously. Collection, storage and use of patient‐derived tissue and data were performed in compliance with the Code for Proper Secondary Use of Human Tissue in the Netherlands, Dutch Federation of Biomedical Scientific Societies, the Netherlands.

### Diagnosis

Tumour marker levels of CEA and CA19‐9 were recorded for all patients before CRS–HIPEC and repeat CRS–HIPEC. CA125 level was recorded if available. CEA level was considered raised when 6 μg/l or above, CA19‐9 level when 37 kunits/l or above, and CA125 level when 20 kunits/l or more. Subgroups were constructed for CEA and CA19‐9 based on tumour marker levels three times higher than the laboratory reference range. CEA was categorized as follows: no increase, less than 6 μg/l; increase threefold or less, 6–20 μg/l; increase more than threefold, above 20 μg/l. CA19‐9 was categorized as: no increase, less than 37 kunits/l; increase less than threefold or less 37–100 kunits/l; increase more than threefold, above 100 kunits/l.

PMP was originally classified as DPAM, PMCA or PMCA‐I, according to Ronnett and colleagues^18^. For the present analysis, patients were reclassified as having acellular mucin, DPAM or PMCA according to the 2016 consensus paper of the Peritoneal Surface Oncology Group International group^16^: acellular mucin is described as mucin without epithelial cells, DPAM as PMP with low‐grade histological features, and PMCA as PMP with high‐grade histological features. Patients with acellular mucin were identified based on a review of all pathology reports. Microscopic slides were reassessed when the histopathological PMP classification was unclear or lacking, in all patients previously classified as having PMCA or PMCA‐I, and when the primary tumour location was unclear. Unidentified primary tumours were approached according to Ronnett *et al*.^18^ and classified as appendiceal neoplasms when they fit the criteria^19^ (following appendicectomy in the past or when no other cause was found in the abdomen).

### Surgical treatment

Patients with PMP were treated with CRS–HIPEC after previous debulking surgery. This combined procedure has been described in detail by Verwaal and co‐workers^20^. The previous surgical score, according to Sugarbaker^2^, was assessed in all patients based on the previous debulking surgery^4^. A score of zero indicated diagnosis via biopsy or laparoscopy; for a score of three, the maximum score, complete debulking surgery had been performed. The extent of peritoneal disease was scored using the Dutch region count, which divides the abdomen into seven regions^21^. A region count of zero indicated no peritoneal disease, and seven affected regions implied peritoneal disease in all regions.

The surgical procedure started with the cytoreductive phase, with the goal of leaving no macroscopic peritoneal disease in the abdomen. The completeness of cytoreduction was recorded. R1 resection indicated no visible macroscopic disease. In R2a resections tumour nodules of less than 2·5 mm were left behind, and in R2b resections peritoneal tumour lesions larger than 2·5 mm were left behind in the abdomen. The cytoreductive phase was followed by HIPEC, designed to eliminate microscopic residual disease. The peritoneal cavity was filled with mitomycin C 35 mg/m^2^ at 40–41 °C for 90 min. In exceptional cases (with subsequent CRS–HIPEC procedures), oxaliplatin 460 mg/m^2^ was used intraperitoneally at 42–43 °C for 30 min. After completion of the HIPEC phase, anastomoses were made and stomas created as necessary. After surgery all patients were admitted to the ICU.

Postoperative complications were scored according to the Common Terminology Criteria for Adverse Events (CTCAE) v4.03^22^. Administration of systemic chemotherapy was not standard of care, but was considered appropriate for some patients with PMCA.

### Follow‐up

Patients were scheduled for follow‐up at least annually for a minimum of 10 years after surgery or until death. Follow‐up included serum tumour markers and CT of the abdomen and pelvis annually, for up to 5 years. Thereafter, CT was performed every 2 years and serum tumour markers annually. Recurrences during follow‐up were recorded, including location and treatment. Abnormalities on CT and raised levels of tumour markers at the time of disease recurrence were also recorded.

### Statistical analysis

Patient‐, surgery‐ and tumour‐related data were compared between PMP subgroups. Categorical variables were tested using Pearson's χ^2^ or Fisher's exact test as appropriate, and presented as numbers of patients with percentages. Ordinal and continuous variables were tested using the linear‐by‐linear or Kruskal–Wallis test, and presented as median (i.q.r.) values.

Disease‐free survival (DFS) was defined as time from first CRS–HIPEC procedure to the date of first recurrence, and patients were censored at date of last follow‐up. OS was defined as time from first CRS–HIPEC to date of death, and patients were censored at date of last follow‐up. DFS and OS curves were constructed using the Kaplan–Meier method and compared with the log rank test.

Univariable and multivariable Cox regression analyses were used to investigate prognostic factors for DFS and OS after primary CRS–HIPEC. Factors with *P* ≤ 0·100 in univariable models were included in the multivariable Cox regression model. Proportionality assumptions for all Cox models were checked and fulfilled. Two‐sided *P* < 0·050 was considered statistically significant. Statistical analyses were performed with IBM SPSS® statistics version 22.0 (IBM, Armonk, New York, USA), STATA® version 13 (StataCorp, College Station, Texas, USA) or GraphPad Prism® version 7.03 (GraphPad Software, San Diego, California, USA).

## Results

**Figure 1:**
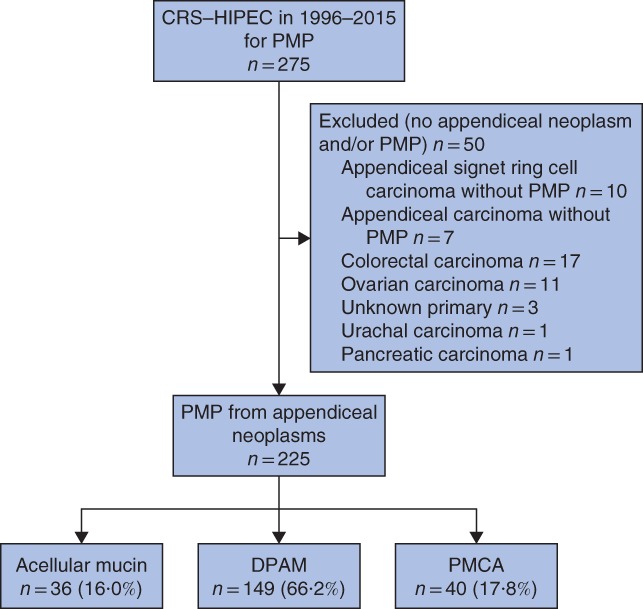
Flow diagram of patient selection. CRS, cytoreductive surgery; HIPEC, hyperthermic intraperitoneal chemotherapy; PMP, pseudomyxoma peritonei; DPAM, disseminated peritoneal adenomucinosis; PMCA, peritoneal mucinous carcinomatosis

Some 225 patients were included (*Fig*. 1). Their baseline characteristics are presented in *Table* 1. Most had undergone previous laparoscopy or laparotomy for PMP. Patients with acellular mucin were more often asymptomatic at time of diagnosis. Serum CEA and CA19‐9 levels, as well as number of affected regions and administration of chemotherapy, differed between the histological groups.

**Table 1:** Baseline characteristics by histological pseudomyxoma peritonei classification based on first cytoreductive surgery–hyperthermic intraperitoneal chemotherapy procedure

		Acellular mucin (*n* = 36)	DPAM (*n* = 149)	PMCA (*n* = 40)	*P* ¶
Age (years)*		58·1 (48·5–70·1)	57·2 (47·7–64·7)	54·5 (46·5–69·2)	0·504#
Sex ratio (M : F)		6 : 30	51 : 98	9 : 31	0·067
ASA fitness grade	I	19 (53)	71 (47·7)	17 (43)	0·861
	II	14 (39)	62 (41·6)	20 (50)	
	III	3 (8)	16 (10·7)	3 (8)	
Co‐morbidity	No	13 (36·1)	73 (49·0)	22 (55)	0·237
	Yes	23 (64)	76 (51·0)	18 (45)	
Symptoms at presentation†	Asymptomatic	10 (28)	20 (13·4)	1 (3)	0·007
	Abdominal	20 (56)	105 (70·5)	30 (75)	0·081
	Weight loss	1 (3)	19 (12·8)	4 (10)	0·232
	Nausea/vomiting	1 (3)	4 (2·7)	0 (0)	0·661
	Incidental finding‡	10 (28)	24 (16·1)	7 (18)	0·231
Colonoscopy performed before CRS–HIPEC	No	26 (72)	110 (73·8)	24 (60)	0·269
	Yes, no abnormalities	9 (25)	37 (24·8)	16 (40)	
	Yes, suspicious lesions§	1 (3)	2 (1·3)	0 (0)	
No. of operations before CRS–HIPEC	0	7 (19)	30 (20·1)	7 (18)	0·511
	1	25 (69)	94 (63·1)	22 (55)	
	2	4 (11)	20 (13·4)	7 (18)	
	3	0 (0)	4 (2·7)	4 (10)	
	4	0 (0)	1 (0·7)	0 (0)	
Previous surgical score	0	1 (3)	13 (8·7)	5 (13)	0·105
	1	4 (11)	13 (8·7)	3 (8)	
	2	18 (50)	41 (27·5)	11 (28)	
	3	6 (17)	52 (34·9)	14 (35)	
	No previous surgery	7 (19)	30 (20·1)	7 (18)	
Systemic chemotherapy	None	35 (97)	131 (87·9)	24 (60)	< 0·001
	Neoadjuvant	0 (0)	1 (0·7)	2 (5)	
	Adjuvant	1 (3)	17 (11·4)	13 (33)	
	Perioperative	0 (0)	0 (0)	1 (3)	
Peritoneal disease	Synchronous	33 (92)	141 (94·6)	35 (88)	0·243
	Metachronous	3 (8)	8 (5·4)	5 (13)	
Distant metastases (before CRS–HIPEC)	No	36 (100)	148 (99·3)	40 (100)	1·000
	Yes	0 (0)	1 (0·7)	0 (0)	
Marker level before CRS–HIPEC*	CEA (μg/l)	2 (1–3)	12 (3–54)	19 (3–93)	< 0·001#
	CA19‐9 (kunits/l)	10 (6–19)	27 (9–174)	66 (7–341)	0·001#
	CA125 (kunits/l)	12 (7–29)	41 (14–81)	37 (21–74)	0·097#
No. of CRS–HIPEC procedures	1	36 (100)	115 (77·2)	35 (88)	0·007
	2	0 (0)	29 (19·5)	5 (13)	
	3	0 (0)	5 (3·4)	0 (0)	
No. of regions involved	0–2	10 (28)	17 (11·4)	3 (8)	0·003
	3–5	17 (47)	43 (28·9)	13 (33)	
	6–7	9 (25)	86 (57·7)	23 (58)	
	Unknown	0 (0)	3 (2·0)	1 (3)	
Completeness of cytoreduction	R1	28 (78)	93 (62·4)	27 (68)	0·208
	R2a	8 (22)	37 (24·8)	9 (23)	
	R2b	0 (0)	17 (11·4)	4 (10)	
	Unknown	0 (0)	2 (1·3)	0 (0)	
Intraperitoneal chemotherapy	Mitomycin C	35 (97)	149 (100)	40 (100)	0·160
	Oxaliplatin	1 (3)	0 (0)	0 (0)	
CTCAE grade	0	19 (53)	48 (32·2)	13 (33)	0·098
	1–2	9 (25)	29 (19·5)	12 (30)	
	3–4	8 (22)	62 (41·6)	14 (35)	
	5	0 (0)	9 (6·0)	1 (3)	
	Unknown	0 (0)	1 (0·7)	0 (0)	
In‐hospital mortality	No	36 (100)	140 (94·0)	39 (98)	0·313
	Yes	0 (0)	9 (6·0)	1 (3)	

Values in parentheses are percentages unless indicated otherwise;

* values are median (i.q.r.). †Patients could have multiple complaints at time of presentation;

‡ patients could have an appendicitis, be suspected of having ovarian cancer, or have an inguinal or umbilical hernia;

§ appendiceal volcano sign, granular aspect caecum and T1 adenocarcinoma sigmoid.

DPAM, disseminated peritoneal adenomucinosis; PMCA, peritoneal mucinous carcinomatosis; CRS, cytoreducive surgery; HIPEC, hyperthermic intraperitoneal chemotherapy; CEA, carcinoembryonic antigen; CA, carbohydrate/cancer antigen; CTCAE, Common Terminology Criteria for Adverse Events^22^.

¶ χ^2^ or Fisher's exact test, except #Kruskal–Wallis test.

### Co‐morbidity related to CRS–HIPEC

Eighty patients (35·6 per cent) did not develop postoperative complications after their first CRS–HIPEC procedure. Ten patients (4·4 per cent) died from complications, five (2·2 per cent) within 30 days. Postoperative complication rates after second and third CRS–HIPEC procedures were comparable (*Table* S1, supporting information).

Two patients (0·9 per cent) had a stoma before the first CRS–HIPEC procedure, and afterwards 79 patients (35·1 per cent) had an ileostomy or colostomy (*Table* S1, supporting information). Overall stoma reversal was possible in 16 of 79 patients (20 per cent). After a second CRS–HIPEC procedure in 39 patients, a stoma was created in 19 (49 per cent). Bowel continuity was restored in none of these patients, and four of five patients had a permanent stoma after a third CRS–HIPEC procedure (*Table* S1, supporting information).

### Management of recurrences

Of 36 patients diagnosed with acellular mucin, seven (19 per cent) had locoregional recurrence of disease. One of these patients had undergone R2a resection. In patients with DPAM, systemic recurrences, a combination of locoregional and systemic recurrences, and locoregional recurrences were observed in two (1·3 per cent), five (3·4 per cent) and 81 patients (54·4 per cent) respectively. Of 17 patients diagnosed with DPAM who had an R2b resection, four did not show recurrence of disease during follow‐up. After CRS–HIPEC for PMCA, systemic recurrences, a combination of locoregional and systemic recurrences, and locoregional recurrences were observed in one (3 per cent), five (13 per cent) and 19 patients (48 per cent) respectively. Additional data regarding locations of recurrence are shown in *Table* S2 (supporting information).

**Figure 2:**
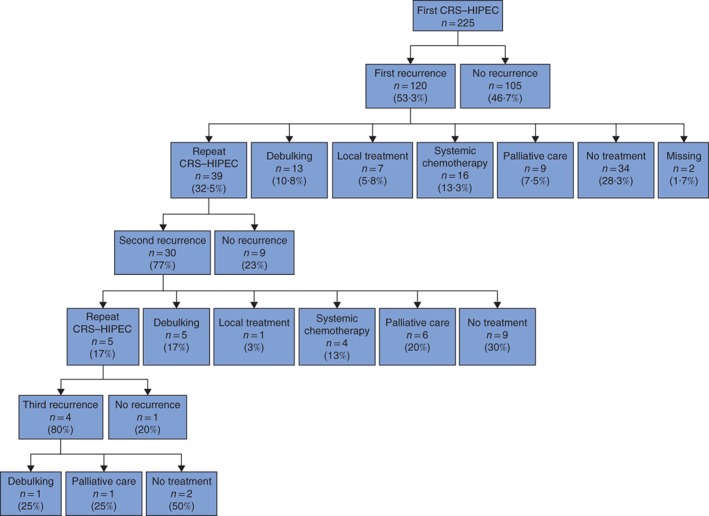
Management of peritoneal recurrence in patients with pseudomyxoma peritonei. No treatment was given in 34 patients (28·3 per cent) after first recurrence: watchful waiting (21 patients), poor physical condition (due to postoperative complications) (5), no available treatment options (5) or other reason (3)

The management of PMP recurrences is shown in *Fig*. 2. After the first CRS–HIPEC procedure, 105 patients (46·7 per cent) remained disease‐free after a median of 68·8 (i.q.r. 27·1–125·7) months of follow‐up. Recurrences were diagnosed in the remaining 120 patients (53·3 per cent) after a median of 16·3 (8·1–41·1) months. Patients with recurrence had a median OS of 85·2 (38·4–171·6) months. When recurrence developed after the first CRS–HIPEC procedure, 39 patients (32·5 per cent) underwent a second course of CRS–HIPEC (*Fig*. 2). Thirty (77 per cent) of these 39 patients developed further recurrences at a median survival of 59·4 (32·1–124·5) months. For the nine patients with no recurrence after a second CRS–HIPEC procedure, median survival was not reached. Five patients (17 per cent) treated with a third CRS–HIPEC procedure had a median OS of 23·5 (21·5 to infinity) months. The other treatment options for recurrences and extrasurgical treatments are shown in *Fig*. 2.

### Survival

**Figure 3:**
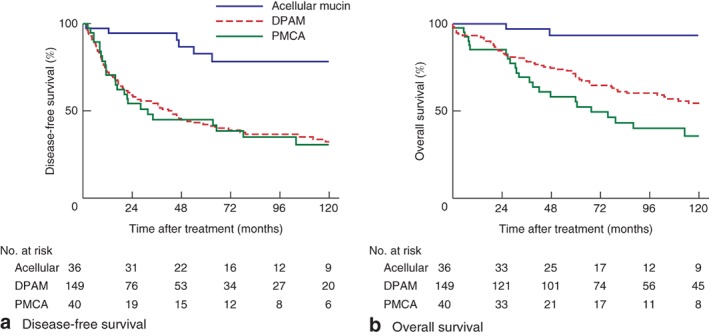
Kaplan–Meier analysis of survival in 225 patients with pseudomyxoma peritonei by histological classification. DPAM, disseminated peritoneal adenomucinosis; PMCA, peritoneal mucinous carcinomatosis. **a,b** *P* < 0·001 (log rank test)

Median follow‐up after the first CRS–HIPEC procedure was 68·1 (i.q.r. 32·7–124·2) months. Median DFS until first recurrence was significantly longer in patients with acellular mucin (median DFS not reached) compared with that in patients with DPAM (41·9 (10·6–207·9) months) or PMCA (28·1 (11·1 to infinity) months (*Fig*. 3 *a*). Factors associated with poor DFS in multivariable analysis were six or seven affected regions (hazard ratio (HR) 6·01, 95 per cent c.i. 2·04 to 17·73), incomplete cytoreduction (R2a resection: HR 1·67, 1·05 to 2·65; R2b resection: HR 2·00, 1·07 to 3·73), and more than threefold increased CEA and/or CA19‐9 level (HR 2·31, 1·30 to 4·11) (*Table* 2).

**Table 2:** Univariable and multivariable Cox regression analysis of disease‐free survival in all patients

			Univariable DFS		Multivariable DFS	
		DFS (months)*	Hazard ratio	*P*	Hazard ratio	*P*
Age			0·99 (0·97, 1·00)	0·062		
Sex	M	26·0 (8·3–112·4)	1·85 (1·26, 2·73)	0·002	1·49 (0·94, 2·34)	0·088
	F	78·3 (17·2–207·9)	1·00 (reference)		1·00 (reference)	
ASA grade				0·783		
	I	58·9 (15·6–207·9)	1·00 (reference)			
	II	46·7 (11·1–∞)	1·14 (0·78, 1·67)	0·492		
	III	51·0 (14·3–∞)	1·11 (0·59, 2·06)	0·751		
Abdominal symptoms	No	71·2 (16·6–170·3)	1·00 (reference)			
	Yes	54·1 (11·0–207·9)	1·11 (0·74, 1·68)	0·608		
Weight loss ± loss of appetite	No	64·7 (14·3–∞)	1·00 (reference)		1·00 (reference)	
	Yes	36·8 (5·3–207·9)	1·62 (1·02, 2·59)	0·043	0·76 (0·44, 1·30)	0·309
Nausea or vomiting	No	61·3 (12·2–207·9)	1·00 (reference)			
	Yes	26·0 (11·0–∞)	1·10 (0·35, 3·47)	0·870		
Incidental finding				0·838		
	None	63·0 (11·7–207·9)	1·00 (reference)			
	Hernia inguinalis/umbilicalis	n.a. (11·3–∞)	0·70 (0·28, 1·75)	0·445		
	Ovarian mass	37·1 (11·3–46·9)	1·22 (0·53, 2·79)	0·640		
	Appendicitis	45·7 (28·1–136·9)	1·01 (0·53, 1·95)	0·968		
Timing of CRS–HIPEC	1996–2005	43·3 (11·3–207·9)	1·41 (0·97, 2·05)	0·069	0·88 (0·58, 1·33)	0·537
	2006–2015	n.a. (15·9–∞)	1·00 (reference)		1·00 (reference)	
No. of regions involved				< 0·001		0·001
	0–2	n.a.	1·00 (reference)		1·00 (reference)	
	3–5	136·9 (43·4–∞)	3·62 (1·27, 10·33)	0·016	2·82 (0·96, 8·26)	0·060
	6–7	18·2 (8·3–61·3)	11·12 (4·07, 30·43)	< 0·001	6·01 (2·04, 17·73)	0·001
Completeness of cytoreduction				< 0·001		0·025
	R1	104·1 (24·0–207·9)	1·00 (reference)		1·00 (reference)	
	R2a	28·1 (9·3–∞)	1·77 (1·16, 2·70)	0·008	1·67 (1·05, 2·65)	0·030
	R2b	8·9 (2·3–17·2)	4·44 (2·59, 7·60)	< 0·001	2·00 (1·07, 3·73)	0·029
CTCAE grade				0·017		0·856
	0	104·9 (22·0–∞)	1·00 (reference)		1·00 (reference)	
	1–2	65·3 (17·2–∞)	1·05 (0·63, 1·76)	0·840	0·77 (0·43, 1·39)	0·385
	3–4	28·2 (9·0–120·8)	1·84 (1·22, 2·78)	0·004	0·89 (0·51, 1·54)	0·674
	5	n.a.	n.a.	n.a.	n.a.	n.a.
Histological classification of PMP				0·001		0·184
	Acellular mucin	n.a. (136·9–∞)	1·00 (reference)		1·00 (reference)	
	DPAM	41·9 (10·6–207·9)	4·47 (2·07, 9·66)	< 0·001	2·21 (0·95, 5·13)	0·066
	PMCA	28·1 (11·1–∞)	4·65 (2·01, 10·77)	< 0·001	2·06 (0·79, 5·34)	0·139
Peritoneal disease	Synchronous	61·3 (14·3–207·9)	1·00 (reference)		1·00 (reference)	
	Metachronous	16·0 (9·3–63·4)	1·83 (1·05, 3·20)	0·034	2·02 (0·95, 4·31)	0·068
No. of raised tumour markers (before first CRS–HIPEC)†				< 0·001		0·005
	0	170·3 (48·3–∞)	1·00 (reference)		1·00 (reference)	
	1–2 raised threefold or less	64·7 (45·7–∞)	1·54 (0·83, 2·86)	0·167	1·09 (0·54, 2·19)	0·815
	1–2 raised more than threefold	16·7 (7·4–61·3)	4·31 (2·78, 6·68)	< 0·001	2·31 (1·30, 4·11)	0·004
Systemic chemotherapy	No	65·3 (15·9–207·9)	1·00 (reference)		1·00 (reference)	
	Yes	20·2 (8·3–120·8)	1·66 (1·07, 2·56)	0·024	1·38 (0·78, 2·43)	0·269

Values in parentheses are 95 per cent c.i. unless indicated otherwise;

* values are median (i.q.r.).

† Tumour markers: carcinoembryonic antigen and carbohydrate antigen 19‐9. DFS, disease‐free survival; n.a., not applicable (median survival not reached); CRS, cytoreductive surgery; HIPEC, hyperthermic intraperitoneal chemotherapy; CTCAE, Common Terminology Criteria for Adverse Events^22^; PMP, pseudomyxoma peritonei; DPAM, disseminated peritoneal adenomucinosis; PMCA, peritoneal mucinous carcinomatosis.

Median OS was not reached in patients with acellular mucin, whereas median OS for patients with DPAM was 130·8 (i.q.r. 47·7–201·6) months and that in patients with PMCA was 67·6 (30·7 to infinity) months (*Fig*. 3 *b*). OS rates after 3, 5 and 10 years were 97, 93 and 93 per cent for acellular mucin, 78·1, 69·8 and 54·5 per cent for DPAM, and 69, 55 and 36 for PMCA. Two patients were lost to follow‐up after 5 years. Factors associated with worse OS were male sex (HR 1·74, 95 per cent c.i. 1·09 to 2·77), incomplete cytoreduction (R2a resection: HR 1·87, 1·14 to 3·08; R2b resection: HR 2·28, 1·19 to 4·34), and greater than threefold raised CEA and/or CA19‐9 level (HR 2·89, 1·36 to 6·16) (*Table* 3).

**Table 3:** Unvariable and multivariable Cox regression analysis of overall survival in all patients

			Univariable OS		Multivariable OS	
		OS (months)*	Hazard ratio	*P*	Hazard ratio	*P*
Age			1·01 (0·99, 1·03)	0·238		
Sex	M	78·9 (25·3–171·6)	2·15 (1·43, 3·24)	< 0·001	1·74 (1·09, 2·77)	0·020
	F	161·4 (63·0–217·5)	1·00 (reference)		1·00 (reference)	
ASA grade				0·002		0·077
	I	201·6 (77·4–∞)	1·00 (reference)		1·00 (reference)	
	II	79·6 (31·4–200·5)	2·19 (1·42, 3·37)	< 0·001	1·54 (0·91, 2·49)	0·113
	III	124·8 (35·8–191·9)	1·72 (0·89, 3·32)	0·106	0·75 (0·35, 1·59)	0·450
Abdominal symptoms	No	160·6 (85·2–217·5)	1·00 (reference)		1·00 (reference)	
	Yes	124·8 (36·1–∞)	1·72 (1·04, 2·86)	0·035	1·00 (0·56, 1·80)	0·999
Weight loss ± loss of appetite	No	160·6 (58·2–217·5)	1·00 (reference)		1·00 (reference)	
	Yes	78·9 (27·8–171·6)	1·83 (1·14, 2·94)	0·012	1·18 (0·69, 2·02)	0·555
Nausea or vomiting	No	132·2 (47·7–217·5)	1·00 (reference)			
	Yes	n.a. (47·7–∞)	0·96 (0·24, 3·91)	0·958		
Incidental finding				0·757		
	None	137·1 (42·1–217·5)	1·00 (reference)			
	Hernia inguinalis/umbilicalis	160·6 (85·2–∞)	0·66 (0·24, 1·82)	0·425		
	Ovarian mass	61·9 (50·8–∞)	1·29 (0·52, 3·20)	0·588		
	Appendicitis	121·6 (78·9–∞)	0·83 (0·38, 1·81)	0·646		
Timing of CRS–HIPEC				0·142		0·941
	1996–2005	127·3 (37·5–217·5)	1·40 (0·89, 2·21)		0·98 (0·60, 1·61)	
	2006–2015	n.a. (61·6–∞)	1·00 (reference)		1·00 (reference)	
No. of regions involved				< 0·001		0·136
	0–2	n.a.	1·00 (reference)		1·00 (reference)	
	3–5	200·5 (88·2–200·5)	5·21 (1·22, 22·30)	0·026	2·93 (0·66, 13·08)	0·158
	6–7	78·9 (30·7–191·9)	12·93 (3·17, 52·76)	< 0·001	4·05 (0·93, 17·71)	0·063
Completeness of cytoreduction				< 0·001		0·010
	R1	201·6 (75·9–∞)	1·00 (reference)		1·00 (reference)	
	R2a	103·2 (34·9–191·9)	2·03 (1·28, 3·22)	0·003	1·87 (1·14, 3·08)	0·014
	R2b	31·1 (15·9–67·6)	5·10 (2·94, 8·87)	< 0·001	2·28 (1·19, 4·34)	0·012
CTCAE grade				1·000		
	0	n.a. (121·6–∞)	1·00 (reference)			
	1–2	160·6 (60·6–∞)	1·00 (0·59, 1·71)	1·000		
	3–4	110·2 (39·2–201·6)	1·00 (0·64, 1·57)	1·000		
	5	0·7 (0·3–1·4)	1·00 (0·02, 47·33)	1·000		
Histological classification of PMP				0·002		0·095
	Acellular mucin	n.a.	1·00 (reference)		1·00 (reference)	
	DPAM	130·8 (47·7–201·6)	8·81 (2·16, 35·91)	0·002	3·04 (0·70, 13·30)	0·139
	PMCA	67·6 (30·7–∞)	12·68 (2·99, 53·83)	0·001	4·61 (0·99, 21·59)	0·052
Peritoneal disease	Synchronous	137·1 (50·8–217·5)	1·00 (reference)			
	Metachronous	138·1 (42·1–171·6)	1·09 (0·56, 2·10)	0·799		
No. of raised tumour markers (before first CRS–HIPEC)†				< 0·001		0·009
	0	201·6 (200·5–∞)	1·00 (reference)		1·00 (reference)	
	1–2 raised threefold or less	n.a. (121·6–∞)	1·31 (0·58, 2·96)	0·523	1·33 (0·54, 3·30)	0·540
	1–2 raised more than threefold	61·9 (24·3–138·1)	4·91 (2·85, 8·43)	< 0·001	2·89 (1·36, 6·16)	0·006
Systemic chemotherapy	No	160·6 (58·2–217·5)	1·00 (reference)			
	Yes	115·3 (38·4–∞)	1·30 (0·80, 2·11)	0·289		

Values in parentheses are 95 per cent c.i. unless indicated otherwise;

* values are median (i.q.r.).

† Tumour markers: carcinoembryonic antigen and carbohydrate antigen 19‐9. OS, overall survival; n.a., not applicable (median survival not reached); CRS, cytoreductive surgery; HIPEC, hyperthermic intraperitoneal chemotherapy; CTCAE, Common Terminology Criteria for Adverse Events^22^; PMP, pseudomyxoma peritonei; DPAM, disseminated peritoneal adenomucinosis; PMCA, peritoneal mucinous carcinomatosis.

### Serum tumour markers

CEA level before CRS–HIPEC was increased in four (11 per cent), 98 (65·8 per cent) and 25 patients (63 per cent) with acellular mucin, DPAM and PMCA respectively. CA19·9 level was raised in no patient, 66 (44·3 per cent) and 21 patients (53 per cent) with acellular mucin, DPAM and PMCA respectively.

**Figure 4:**
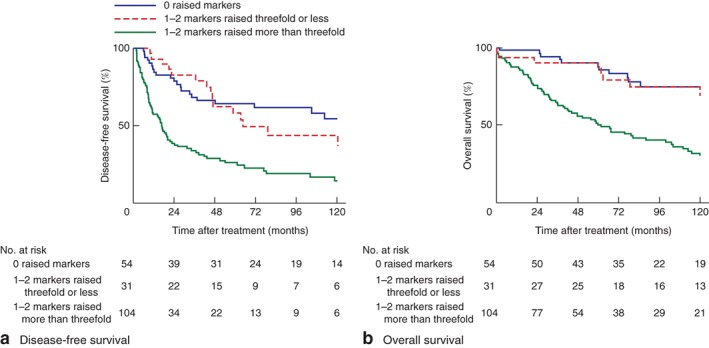
Kaplan–Meier analysis of survival in relation to levels of tumour markers in 189 patients with disseminated peritoneal adenomucinosis or peritoneal mucinous carcinomatosis. **a** Disease‐free and **b** overall survival. Tumour markers: carcinoembryonic antigen or carbohydrate antigen 19‐9. **a,b** *P* < 0·001 (log rank test)

Of patients with DPAM and PMCA, DFS was significantly shorter in those with one or two markers raised more than threefold (median 16·7 (i.q.r. 7·4–61·3) months) compared with that in patients with markers raised threefold or less (64·7 (45·7 to infinity) months) or those with no increased tumour markers (170·3 (48·3 to infinity) months) (*Fig*. 4 *a*).

OS was shorter in patients with DPAM and PMCA with one or two tumour markers raised more than threefold (median 61·9 (24·1–138·1) months) than in patients with one or two tumour markers increased by threefold or less (median OS not reached) or those with no increased markers (201·6 (200·5 to infinity) months) (*Fig*. 4 *b*).

### Timing of CRS–HIPEC

Significant differences were observed between patients who were operated on between 1996 and 2005 and those who had the procedure between 2006 and 2015. In the later cohort, patients were less often treated with systemic chemotherapy, fewer postoperative complications were observed, and ICU and hospital stay were significantly shorter (*Table* S3, supporting information). Acellular mucin was more often diagnosed and fewer patients had recurrence of disease in the later period. The 5‐year OS rate was 66·0 per cent in the 1996–2005 cohort and 76·2 per cent in the 2006–2015 cohort.

## Discussion

Patients with the acellular mucin histological subtype of PMP have an excellent prognosis after CRS–HIPEC. Serum levels of CA19‐9 and CEA were independent prognostic factors, together with sex, number of affected regions and completeness of cytoreduction. CRS–HIPEC procedures were safe in these selected patients. Almost half of the patients with PMP remained disease‐free after treatment with CRS–HIPEC.

The prevalence of histological subtypes for PMP is comparable with that in the literature^16^, ^17^, ^23^. Acellular mucin is associated with the best prognosis, with a 5‐year survival rate of 93 per cent, compared with 69·8 per cent for DPAM and 55 per cent for PMCA. Similar survival rates have been described elsewhere^17^, ^24^. Several other studies^11^, ^25^, ^26^, ^27^ focused on repeat CRS–HIPEC procedures and concluded that this was feasible, with a large group of patients remaining disease‐free afterwards. Recurrence rates after first CRS–HIPEC in the present series were slightly higher than those in other studies, with reported recurrence rates tending to be below 50 per cent^3^, ^11^, ^28^.

Predictors of poorer survival identified in this study have been described previously^28^, ^29^, ^30^, ^31^. Male sex is a poor prognostic factor for survival, although the difference between the sexes remains unexplained^5^, ^29^, ^30^, ^31^. Raised levels of tumour markers have been associated with poorer survival in other studies^1^, ^32^, ^33^. In the present study, increased marker levels were associated with worse prognosis, in particular when CEA was above 20 μg/l and/or CA19‐9 was more than 100 kunits/l. Compared with histological subtype, serum tumour markers are easily determined and might therefore be used to guide the timing of CRS–HIPEC, counsel patients and tailor follow‐up. Levels of these tumour markers were increased only sporadically in patients with acellular mucin, possibly reflecting the absence of tumour cells in the mucinous peritoneal deposits. In DPAM and PMCA, tumour marker levels raised more than threefold were stronger predictors of worse prognosis than histological subtype. A substantial number of patients with PMCA were treated with adjuvant systemic chemotherapy on the basis that in the majority of patients the condition arises from mucinous appendiceal adenocarcinomas, with risk of systemic recurrence.

Bias is inherently present in this study owing to its observational nature, the inclusion of patients who underwent CRS–HIPEC and the long study interval. Time plays an important role, as surgical teams and protocols have changed over the years, with improvements in surgical technique and perioperative care. Definitions and awareness of PMP have also changed during this interval, and pathologists have gained greater experience in recognizing this rare disease with its histopathological subtypes. This could also explain the increase of patients diagnosed with acellular mucin over time.

No RCTs have been performed because PMP is a rare disease, although this might be overcome by greater international collaborative efforts. Until then, observational data remain useful in highlighting management difficulties and pointing the way to worthwhile prospective studies.

## Supporting information


**Table S1** Treatment characteristics and related morbidity by first, second and third CRS–HIPEC procedures
**Table S2** Recurrences, tumour marker levels and imaging abnormalities after first, second and third CRS–HIPEC procedures
**Table S3** CRS–HIPEC by time period (first procedure)
