# Supplementary material for: Factors influencing long‐term survival after cytoreductive surgery and hyperthermic intraperitoneal chemotherapy for pseudomyxoma peritonei originating from appendiceal neoplasms
Source: BJS Open. 2019 Feb 19;3(3):376–86. doi: 10.1002/bjs5.50134 (PMC6551418; doi:10.1002/bjs5.50134)
Supplement: Supplementary file 1 — Table S1 Treatment characteristics and related morbidity by first, second and third CRS–HIPEC procedures Table S2 Recurrences, tumour marker levels and imaging abnormalities after first, second and third CRS–HIPEC procedures Table S3 CRS–HIPEC by time period (first procedure) [file BJS5-3-376-s001.docx]

**BJS5_50120**

**Factors influencing long-term survival after cytoreductive surgery and hyperthermic intraperitoneal chemotherapy for pseudomyxoma peritonei originating from appendiceal neoplasms**

**W. J. van Eden, N. F. M. Kok, P. Snaebjornsson, K. Jόźwiak, K. Woensdregt, P. Bottenberg, H. Boot and A. G. J. Aalbers**

| **Table S1 Treatment characteristics and related morbidity by first, second and third CRS–HIPEC procedures** | | | | | |  |
| --- | --- | --- | --- | --- | --- | --- |
|  | **1st CRS-HIPEC n = 225** | **%** | **2nd CRS-HIPEC n = 39** | **%** | **3rd CRS-HIPEC n = 5** | **%** |
|  |  |  |  |  |  |  |
| **Region count** |  |  |  |  |  |  |
| 0-2 regions | 30 | 13.3% | 7 | 17.9% | 3 | 60.0% |
| 3-5 regions | 73 | 32.4% | 17 | 43.6% | 2 | 40.0% |
| 6-7 regions | 118 | 52.4% | 13 | 33.3% | 0 | 0.0% |
| Unknown | 4 | 1.8% | 2 | 5.1% | 0 | 0.0% |
| **Completeness of cytoreduction** |  |  |  |  |  |  |
| R1 | 148 | 65.8% | 20 | 51.3% | 4 | 80.0% |
| R2a | 54 | 24.0% | 12 | 30.8% | 0 | 0.0% |
| R2b | 21 | 9.3% | 7 | 17.9% | 1 | 20.0% |
| Unknown | 2 | 0.9% | 0 | 0.0% | 0 | 0.0% |
| **Intraperitoneal chemotherapy** |  |  |  |  |  |  |
| No | 0 | 0.0% | 3 | 7.7% | 0 | 0.0% |
| MMC | 224 | 99.6% | 19 | 48.7% | 3 | 60.0% |
| Oxaliplatin | 1 | 0.4% | 16 | 41.0% | 2 | 40.0% |
| **CEA level (prior to CRS-HIPEC)** | 8 (2-44) | - | 14 (6-26) | - | 10 (6-143) | - |
| **CA19.9 level (prior to CRS-HIPEC)** | 23 (8-153) | - | 46 (19-142) | - | 41 (13-43) | - |
| **CA125 level (prior to CRS-HIPEC)** | 36 (13-74) | - | 25 (18-NA) | - | NA | - |
| **Ostomy prior to CRS-HIPEC** |  |  |  |  |  |  |
| No | 223 | 99.1% | 25 | 64.1% | 2 | 40.0% |
| Colostomy | 0 | 0.0% | 14 | 35.9% | 3 | 60.0% |
| Ileostomy | 2 | 0.9% | 0 | 0.0% | 0 | 0.0% |
| **Ostomy (after CRS-HIPEC)** |  |  |  |  |  |  |
| No | 146 | 64.9% | 20 | 51.3% | 1 | 20.0% |
| Colostomy | 73 | 32.4% | 19 | 48.7% | 4 | 80.0% |
| Ileostomy | 6 | 2.7% | 0 | 0.0% | 0 | 0.0% |
| **Serious Adverse Events** |  |  |  |  |  |  |
| Grade 0 | 80 | 35.6% | 14 | 35.9% | 1 | 20.0% |
| Grade 1-2 | 50 | 22.2% | 9 | 23.1% | 3 | 60.0% |
| Grade 3 | 36 | 16.0% | 4 | 10.3% | 0 | 0.0% |
| Grade 4 | 48 | 21.3% | 8 | 20.5% | 1 | 20.0% |
| Grade 5 | 10 | 4.4% | 3 | 7.7% | 0 | 0.0% |
| Unknown | 1 | 0.4% | 1 | 2.6% | 0 | 0.0% |
| **In-hospital mortality** |  |  |  |  |  |  |
| No | 215 | 95.6% | 36 | 92.3% | 5 | 100.0% |
| Yes | 10 | 4.4% | 3 | 7.7% | 0 | 0.0% |
| *30-day mortality* | *5* | *50.0%* | *2* | *66.6%* | *0* | *0.0%* |
| *60-day mortality* | *4* | *40.0%* | *1* | *33.3%* | *0* | *0.0%* |
| *>60-day mortality* | *1* | *10.0%* | *0* | *0.0%* | *0* | *0.0%* |
| **ICU stay (days)** | 3 (2-5) | - | 2 (1-4) | - | 2 (2-4) | - |
| **Hospital stay (days)** | 20 (14-31) | - | 17 (13-26) | - | 17 (11-39) | - |
| **Time between 1st and 2nd HIPEC (months)** | NA | - | 53.4 (23.2-73.1) | - | NA | - |
| **Time between 2nd and 3rd HIPEC (months)** | NA | - | NA | - | 49.0 (33.3-73.5) | - |
| **Legend.** Treatment characteristics of the first, second and third CRS-HIPEC procedures. Categorical data are presented as numbers with percentages and continuous data as medians with interquartile ranges. **Abbreviations.** CRS-HIPEC, cytoreductive surgery and hyperthermic intraperitoneal chemotherapy; ICU, intensive care unit. | | | | | | |
|  |  |  |  |  |  |  |
|  |  |  |  |  |  |  |
|  |  |  |  |  |  |  |

| **Table S2 Recurrences, tumour marker levels and imaging abnormalities after first, second and third CRS–HIPEC procedures** | | | | | | |  |
| --- | --- | --- | --- | --- | --- | --- | --- |
|  | **Acellular mucin** | **%** | **DPAM** | **%** | **PMCA** | **%** | **P-value** |
|  |  |  |  |  |  |  |  |
| **Follow-up after 1st CRS-HIPEC (n = 225)** | **n = 36** |  | **n = 149** |  | **n = 40** |  |  |
| **Location of recurrence** |  |  |  |  |  |  | **<0.001** |
| No recurrence | 29 | 80.6% | 61 | 40.9% | 15 | 37.5% |  |
| Locoregional | 7 | 19.4% | 81 | 54.4% | 19 | 47.5% |  |
| Systemic | 0 | 0.0% | 2 | 1.3% | 1 | 2.5% |  |
| Locoregional + systemic | 0 | 0.0% | 5 | 3.4% | 5 | 12.5% |  |
| **Tumor marker elevation after CRS-HIPEC** | |  |  |  |  |  | **<0.001** |
| No | 35 | 97.2% | 83 | 55.7% | 19 | 48.7% |  |
| Yes | 1 | 2.8% | 66 | 44.3% | 20 | 51.3% |  |
| **Imaging abnormalities after CRS-HIPEC*** |  |  |  |  |  |  | **<0.001** |
| No | 30 | 83.3% | 64 | 43.0% | 16 | 41.0% |  |
| Yes | 6 | 16.7% | 85 | 57.0% | 23 | 59.0% |  |
|  |  |  |  |  |  |  |  |
| **Follow-up after 2nd CRS-HIPEC (n = 39)** | **n = 0** |  | **n = 34** |  | **n = 5** |  |  |
| **Location of recurrence** |  |  |  |  |  |  | 1.000 |
| No recurrence | - | - | 8 | 23.5% | 1 | 20.0% |  |
| Locoregional | - | - | 23 | 67.6% | 4 | 80.0% |  |
| Systemic | - | - | 1 | 2.9% | 0 | 0.0% |  |
| Locoregional + systemic | - | - | 2 | 5.9% | 0 | 0.0% |  |
| **Tumor marker elevation after CRS-HIPEC** | |  |  |  |  |  | 1.000 |
| No | - | - | 12 | 37.5% | 2 | 40.0% |  |
| Yes | - | - | 20 | 62.5% | 3 | 60.0% |  |
| **Imaging abnormalities after CRS-HIPEC*** |  |  |  |  |  |  | 0.609 |
| No | - | - | 9 | 26.5% | 2 | 40.0% |  |
| Yes | - | - | 25 | 73.5% | 3 | 60.0% |  |
|  |  |  |  |  |  |  |  |
| **Follow-up after 3rd CRS-HIPEC (n = 5)** | **n = 0** |  | **n = 5** |  | **n = 0** |  |  |
| **Location of recurrence** |  |  |  |  |  |  | - |
| No recurrence | - | - | 1 | 20.0% | - | - |  |
| Locoregional | - | - | 4 | 80.0% | - | - |  |
| Systemic | - | - | 0 | 0.0% | - | - |  |
| Locoregional + systemic | - | - | 0 | 0.0% | - | - |  |
| **Tumor marker elevation after CRS-HIPEC** | |  |  |  |  |  | - |
| No | - | - | 2 | 40.0% | - | - |  |
| Yes | - | - | 3 | 60.0% | - | - |  |
| **Imaging abnormalities after CRS-HIPEC*** |  |  |  |  |  |  | - |
| No | - | - | 1 | 20.0% | - | - |  |
| Yes | - | - | 4 | 80.0% | - | - |  |
| **Legend.** Follow-up data regarding recurrences after first, second and third CRS-HIPEC. Data are presented as numbers with percentages. **Symbols.** *, Abnormalities seen on follow-up CT scans suspicious for recurrence of peritoneal disease. **Abbreviations.** DPAM; disseminated peritoneal adenomucinosis; PMCA, peritoneal mucinous carcinomatosis; CRS, cytoreductive surgery; HIPEC, hyperthermic intraperitoneal chemotherapy. | | | | | | | |
|  |  |  |  |  |  |  |  |
|  |  |  |  |  |  |  |  |
|  |  |  |  |  |  |  |  |

| **Table S3 CRS–HIPEC by time period (first procedure)** | | | |  |  |
| --- | --- | --- | --- | --- | --- |
|  | **1996-2005 n = 115** |  | **2006-2015 n = 110** |  | **P-value** |
|  |  |  |  |  |  |
| **Number of surgeries prior to CRS-HIPEC** |  |  |  |  | 0.606 |
| 0 | 23 | 20.0% | 21 | 19.1% |  |
| 1 | 70 | 60.9% | 71 | 64.5% |  |
| 2 | 15 | 13.0% | 16 | 14.5% |  |
| 3 | 6 | 5.2% | 2 | 1.8% |  |
| 4 | 1 | 0.9% | 0 | 0.0% |  |
| **Prior Surgical Score** |  |  |  |  | **0.003** |
| 0 | 4 | 3.5% | 15 | 13.6% |  |
| 1 | 8 | 7.0% | 12 | 10.9% |  |
| 2 | 33 | 28.7% | 37 | 33.6% |  |
| 3 | 47 | 40.9% | 25 | 22.7% |  |
| No previous surgery | 23 | 20.0% | 21 | 19.1% |  |
| **Systemic chemotherapy** |  |  |  |  | **0.008** |
| No CTx | 90 | 78.3% | 100 | 90.9% |  |
| Neoadjuvant CTx | 1 | 0.9% | 2 | 1.8% |  |
| Adjuvant CTx | 23 | 20.0% | 8 | 7.3% |  |
| Perioperative CTx | 1 | 0.9% | 0 | 0.0% |  |
| **Region count** |  |  |  |  | **0.049** |
| 0-2 | 9 | 7.8% | 21 | 19.1% |  |
| 3-5 | 37 | 32.2% | 36 | 32.7% |  |
| 6-7 | 65 | 56.5% | 53 | 48.2% |  |
| Unknown | 4 | 3.5% | 0 | 0.0% |  |
| **Completeness of cytoreduction** |  |  |  |  | 0.639 |
| R1 | 78 | 67.8% | 70 | 63.6% |  |
| R2a | 26 | 22.6% | 28 | 25.5% |  |
| R2b | 9 | 7.8% | 12 | 10.9% |  |
| Unknown | 2 | 1.7% | 0 | 0.0% |  |
| **Serious Adverse Events** |  |  |  |  | **0.003** |
| No | 30 | 26.1% | 50 | 45.5% |  |
| Yes | 84 | 73.0% | 60 | 54.5% |  |
| Unknown | 1 | 0.9% | 0 | 0.0% |  |
| **In-hospital mortality** |  |  |  |  | **0.019** |
| No | 106 | 92.2% | 109 | 99.1% |  |
| Yes | 9 | 7.8% | 1 | 0.9% |  |
| **ICU stay (days)** | 5 (3-6) | - | 2 (1-4) | - | **<0.001** |
| **Hospital stay (days)** | 21 (17-35) | - | 16 (12-25) | - | **<0.001** |
| **Histological PMP classification** |  |  |  |  | **<0.001** |
| Acellular mucin | 8 | 7.0% | 28 | 25.5% |  |
| DPAM | 81 | 70.4% | 68 | 61.8% |  |
| PMCA | 26 | 22.6% | 14 | 12.7% |  |
| **Recurrence** |  |  |  |  | **0.002** |
| No | 42 | 36.5% | 63 | 57.3% |  |
| Yes | 73 | 63.5% | 47 | 42.7% |  |
| **Legend.** Chacteristics of the first CRS-HIPEC procedure in two time periods: 1996 to 2005 and 2006 to 2015. **Abbreviations.** CRS, cytoreductive surgery; HIPEC, hyperthermic intraperitoneal chemotherapy; ICU, intensive care unit; DPAM, disseminated peritoneal adenomucinosis; PMCA; peritoneal mucinous carcinomatosis. | | | | | |
|  |  |  |  |  |  |
|  |  |  |  |  |  |
|  |  |  |  |  |  |
|  |  |  |  |  |  |
